# PReS-FINAL-2038: Clinical efficacy and tolerability of tocilizumab in patients with persistant systemic JIA

**DOI:** 10.1186/1546-0096-11-S2-P51

**Published:** 2013-12-05

**Authors:** D Mihailova, B Varbanova, S Stefanov, A Teltcharova

**Affiliations:** 1 Pediatric Clinic of Rheumatology, Cardiology and Hematology, University Pediatric Hospital, Medical University, Sofia, Bulgaria; 2 Medical University of Varna, University Hospital St.Anna-Varna, Pediatric Clinic, Varna, Bulgaria; 3 Pediatric Clinic of Rheumatology, Cardiology and Hematology, University Pediatric Hospital, Medical University, Sofia, Bulgaria, Sofia, Bulgaria

## Introduction

Tocilizumab was recommended for the treatment of systemic juvenile idiopathic arthritis (sJIA) in children whose disease has responded inadequately to non-steroidal anti-inflammatory drugs, systemic corticosteroids and methotrexate.

## Objectives

To study the efficacy and tolerability of Tocilizumab (TCZ) in patients with longstanding refractory to the conventional treatment and TNF-inhibitors sJIA.

## Methods

Seven children in the age range 2-12 years (mean 6.3 years) with disease duration 1.2 - 9 years (mean 4.6 years) with active persistent sJIA have been enrolled in one-year prospective study. All 7 patients had oligoarticular joint involvement and received previous treatment with NSAIDS, Methotrexate and corticosteroids. 4/7 had concomitant therapy with Etanercept and 2/7 second DMARD. In all patients TCZ was administered immediately after discontinuation of the biological treatment or the second DMARD in a dose of 8 mg/kg for patients ≥ 30 kg and 12 mg/kg for patients < 30 kg every two weeks. At baseline all patients had active disease. Response to the treatment was measured according to the ACR Pedi criteria at the end of the 1st, 3rd, 6^th^, 9^th ^and 12^th ^month.

Clinical examination, laboratory investigations, growth parameters' assessment and screening for adverse events have been performed every 2 weeks.

## Results

At the end of the 1^st ^month we found complete resolution of the systemic symptoms and acute phase response in all patients. At the end of the 3^rd ^month, ACR Pedi 30, 50, 70 and 90 responses were achieved by 7 (100%), 5 (71%), 1(14%) and 0 (0%) patients, respectively. By 6^th ^month the observed responses we as follows: ACR Pedi 30 - 7 pts (100%), ACR Pedi 50 - 7 pts (100%), ACR Pedi 70 - 5 pts (71%), ACR 90 - 1 patient (14%). At the end of 9^th ^month ACR Pedi 30, 50, 70 and 90 responses were achieved by 7 (100%), 7 (100%), 6 (86%) and 4 (57%) patients. All patients maintained these parameters by 12^th ^month.

After achieving control of the disease activity, we started to reduce the dose of steroids and/or Methotrexate leading to their discontinuation and monotherapy with TCZ in 4 patients. In all of these patients remission on medication was documented by 12^th ^month. At the end of the observational period the linear growth velocity of all treated patients reached the average normal values per year of their age matched peers.

Some patients experienced mild and reversible adverse reactions such as: mild neutropenia or thrombocytopenia, slight increase of liver enzymes and increased cholesterol levels. All side effects resolved with the decrease of the dosage and did not lead to the discontinuation of the treatment.

## Conclusion

Tocilizumab is an effective and well tolerated drug in children with systemic longstanding persistent JIA, refractory to the conventional treatment and TNF-inhibition. Monotherapy with TCZ is an appropriate option for controlling the disease activity, improving the physical growth and minimizing the overall toxicity of the treatment of this disease.

## Disclosure of interest

None declared.

